# Assessing and Mapping of Road Surface Roughness based on GPS and Accelerometer Sensors on Bicycle-Mounted Smartphones

**DOI:** 10.3390/s18030914

**Published:** 2018-03-19

**Authors:** Kaiyue Zang, Jie Shen, Haosheng Huang, Mi Wan, Jiafeng Shi

**Affiliations:** 1 Key Laboratory of Virtual Geographic Environment (Nanjing Normal University), Ministry of Education, Nanjing 210023, China; kaiyuezang@163.com (K.Z.); onemefly@163.com (M.W.); jiafeng.shi@outlook.com (J.S.); 2 School of Geography Sciences, Nanjing Normal University, Nanjing 210023, China; 3 Jiangsu Center for Collaborative Innovation in Geographical Information Resource Development and Application, Nanjing 210023, China; 4 GIScience Center, University of Zurich, 8057 Zurich, Switzerland

**Keywords:** road surface roughness, IRI, bicycle-mounted smartphone, accelerometer, GPS

## Abstract

The surface roughness of roads is an essential road characteristic. Due to the employed carrying platforms (which are often cars), existing measuring methods can only be used for motorable roads. Until now, there has been no effective method for measuring the surface roughness of un-motorable roads, such as pedestrian and bicycle lanes. This hinders many applications related to pedestrians, cyclists and wheelchair users. In recognizing these research gaps, this paper proposes a method for measuring the surface roughness of pedestrian and bicycle lanes based on Global Positioning System (GPS) and accelerometer sensors on bicycle-mounted smartphones. We focus on the International Roughness Index (IRI), as it is the most widely used index for measuring road surface roughness. Specifically, we analyzed a computing model of road surface roughness, derived its parameters with GPS and accelerometers on bicycle-mounted smartphones, and proposed an algorithm to recognize potholes/humps on roads. As a proof of concept, we implemented the proposed method in a mobile application. Three experiments were designed to evaluate the proposed method. The results of the experiments show that the IRI values measured by the proposed method were strongly and positively correlated with those measured by professional instruments. Meanwhile, the proposed algorithm was able to recognize the potholes/humps that the bicycle passed. The proposed method is useful for measuring the surface roughness of roads that are not accessible for professional instruments, such as pedestrian and cycle lanes. This work enables us to further study the feasibility of crowdsourcing road surface roughness with bicycle-mounted smartphones.

## 1. Introduction

The surface roughness of roads is an important road characteristic, and it is closely related to driving safety, passenger comfort, road maintenance and so on. With the increase of road mileage all over the world, the quality of roads has become an important concern. Among different road quality indicators, road roughness is probably one of the most important ones [1,2]. No matter which mode of transportation users take, bumpy roads will significantly increase the difficulty of travel and the potholes/humps of a road will even hinder the progress of vehicles and road users. Moreover, bumpy roads significantly reduce the lifespan of the roads themselves [1]. Therefore, recently, lots of research interest has been centered on developing methods for collecting information regarding pavement [3,4,5].

While existing measuring methods, such as vehicle-mounted 3D LiDAR measuring systems, can provide very accurate information on road surface roughness, they are often very expensive and restricted to professional applications [1]. There are also several studies that have used low-cost accelerometers and other sensors available on smartphones to measure road surface roughness, mainly using cars as the carrying platforms [6,7,8]. However, due to the employed vehicles, these systems can only be used on motorable roads. Until now, there has been no effective method of measuring the road surface roughness of pedestrian and bicycle lanes, which hinders many potential applications related to pedestrians, wheelchair users and cyclists, such as navigation and routing [9,10,11].

At present, smartphones are integrating more and more sensors (e.g., Global Positioning System (GPS) and accelerometers). These sensors can potentially provide some necessary data to estimate road surface roughness. For example, the most commonly used measure of road surface roughness is the International Roughness Index (IRI) [1,12]. To compute IRI values, the accumulative vertical displacement value (resulting from potholes and humps on the road) and the overall travel distance are needed. The accumulative vertical displacement value can be derived using accelerometer sensors, while the overall travel distance can be obtained using GPS sensors. These two kinds of sensors are available in most of the current smartphones. This makes smartphones promising devices for measuring road surface roughness. Meanwhile, due to their rather rigid structure, bicycles are often very sensitive to the ups and downs of roads. Mounting smartphones on bicycles would potentially allow users to map road surface roughness when they travel around, especially on bicycle and pedestrian lanes. This will become a more promising option as cycling in cities becomes more and more popular, which will potentially allow large-scale crowdsourcing of road surface roughness, similar to the famous volunteered geographic information (VGI) project OpenStreetMap (https://www.openstreetmap.org/).

In recognizing the research gaps and the high potential of smartphones, this paper aims to propose a method for measuring the road surface roughness of bicycle and pedestrian lanes based on GPS and accelerometer sensors on bicycle-mounted smartphones. We mainly focus on IRI. Firstly, we analyzed the computing model of road surface roughness and derived the parameters of the IRI model with a bicycle-mounted GPS and accelerometer on a smartphone. An algorithm was then proposed to identify potholes and humps on roads. As a proof of concept, an iOS mobile application “RoadSR” was developed based on the proposed method. Furthermore, three experiments were designed to evaluate the proposed method. We demonstrate that the IRI values measured by the proposed method strongly and positively correlate with those measured by professional instruments.

The rest of this article is organized as follows: Section 2 presents related work. In Section 3, we describe the proposed method. Section 4 reports the proof of concept and the evaluation, and Section 5 discusses the results. We draw conclusions and future work in Section 6.

## 2. Related Work

### 2.1. Road Surface Roughness: Definitions and Evaluation Metrics

Road surface roughness can be understood as an expression of the deviation of the road surface from a true planar surface [13]. It is an important road characteristic because it affects not only riding quality, but also safety, road service life, fuel consumption and maintenance costs [1]. It is often used interchangeably with smoothness. Road surface roughness cannot be measured directly; it requires calculations and deductions based on measurements.

The statistical analysis of road roughness began in the fifties in the twentieth century. In approximately 1950, the American Association of State Highway and Transportation Officials (AASHTO) conducted a subjective evaluation of road roughness [14]. In 1966, Elson Spangler and William Kelly from General Electric developed a first objective measurement method using a longitudinal inertial measuring instrument. The underlying theory behind this method is still in use today [15].

Different evaluation metrics have been proposed to indicate or report road surface roughness, such as the International Roughness Index (IRI), Present Serviceability Rating (PSR), Root Mean Square Vertical Acceleration (RMSVA), Mean Panel Rating (MPR), Ride Number (RN), Slope Variance (SV), and Profile Index (PI) [1,16]. Among them, IRI, which was developed by the World Bank in the 1980s [12], is currently the most widely used indicator of road surface roughness, because it can be transformed to other metrics. It is computed using “a mathematical model known as the ‘quarter-car model’, which represents the way a single tire system (a quarter of a car) is affected by the profile of the pavement [/road]” [1]. The commonly recommended units are meters per kilometer (m/km) or millimeters per meter (mm/m) [17]. Based on IRI measurements, several other indices have been proposed, such as the Mean Ride Index (MRI) and the Half-Car Ride Index (HRI), which have no essential differences to IRI. In this research, we used the IRI to represent road surface roughness.

### 2.2. Measurement Methods of Road Surface Roughness

**Figure 1:**
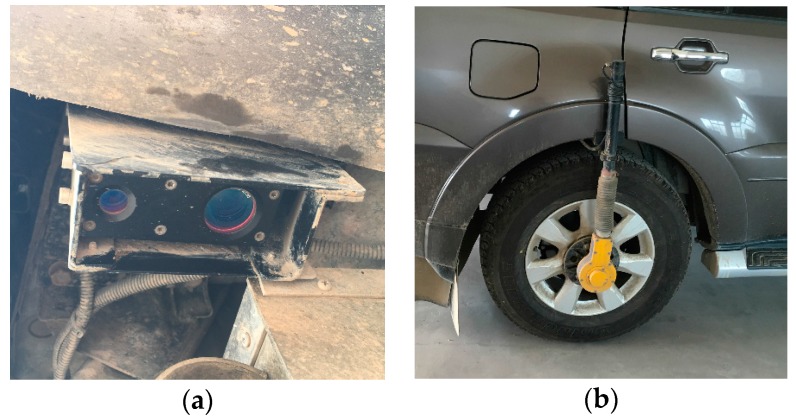
Vehicle-mounted laser cross section measuring system: (**a**) laser Scanner to record the longitudinal displacement (**b**) horizontal displacement recorder.

A number of different professional methods and devices have been used over the years for measuring road surface roughness [1,3,4], including Rod and Level, Straight Edge, Profilograph, Response-Type Instruments, Walking Profilers, and Inertial Profilers. Inertial Profilers are the most sophisticated roadway profiling system currently available. A typical example in regard to this aspect is the vehicle-mounted laser cross section measuring system (Figure 1), which uses a laser scanner and accelerometer to record the longitudinal displacement, and a horizontal displacement recorder to measure the distance travelled. These kinds of systems provide a relatively accurate and repeatable simulation of the road profile, which can then be analyzed to produce various roughness statistics, including the IRI. These systems are often very expensive and restricted to professional applications in highway engineering. More importantly, due to the employed vehicles, these systems can only be used on motorable roads, and are not suitable for measuring the surface roughness of other roads, such as cycle lanes and pedestrian lanes.

With the rapid development in mobile sensor technology, the cost of accelerometers and other measurement sensors (e.g., GPS) is getting lower and lower. These sensors as well as others have become portable and have been integrated into most of the existing smartphones. Therefore, recent research interest has been directed toward using these low-cost accelerometers and other sensors to measure road surface roughness [8]. In the following section, we review these studies.

Most of the existing studies related to this aspect used cars as a carrying platform. For example, Gonzalez et al. (2008) placed a tri-axial accelerometer on a car to obtain acceleration information and estimate the road surface roughness [18]. Eriksson et al. (2008) added a GPS device to Gonzalez et al.’s solution to record trajectories and locate potholes [19]. Mednis et al. (2010) further added a microphone to identify potholes from the sound signal [20]. Vittorio Astarita and colleagues assessed road surface quality based on GPS and accelerometer data on smartphones placed on cars [21,22,23]. Several recent studies also used cars as a carrying platform. Some of them further investigated the relationships between sensor output and the roughness index [7,24,25], while the others proposed algorithms for pothole recognition [26]. Li and Goldberg (2018) proposed a mobile crowdsensing system for road surface assessment based on GPS and accelerometer data from smartphones placed on cars [6]. Alessandroni et al. (2017) further investigated the influence of speed on road roughness sensing using smartphones on cars [27]. Different from the above studies, Tai et al. (2010) used a motorcycle as a carrying tool, and used the smartphone’s accelerometer and GPS sensor to detect road damage location information [28]. Chang et al. (2009) used automatic robots with GPS equipment to estimate road roughness [29].

Due to the fact that IRI and other metrics of road surface roughness cannot be measured directly, a computational model is needed to convert sensor output (e.g., acceleration data from accelerometer sensors) to these metrics. The most direct method is calculating the cumulative vertical displacement value of road bumps by using the quadratic integral of acceleration data [25]. Other attempts have included the artificial neural network [30,31], spectrum analysis [32,33], frequency domain analysis [7], and the genetic algorithm [24]. However, it is also important to note that these studies mainly used cars as the carrying platform.

In summary, several methods have been proposed involving the use of low-cost accelerometers and other sensors to measure road surface roughness and have shown promising results compared to professional equipment. These studies have mainly used cars as the carrying platform, while several others have relied on motorcycles and automatic robots. Due to the carrying platforms employed, these methods are not suitable for measuring the surface roughness of un-motorable roads, such as cycle lanes and pedestrian lanes. Until now, there has been no effective method for measuring the surface roughness of these roads.

In recognizing these research gaps, this research aimed to propose a method for measuring the road surface roughness of pedestrian and cycle lanes. Particularly, we studied the feasibility of using GPS and accelerometer sensors on bicycle-mounted smartphones for assessing road surface roughness.

## 3. Methodology

To comprehensively address the above research objectives, we applied the following methodology.

### 3.1. Computational Model of Road Surface Roughness: IRI

Road surface roughness does not have a unified general definition. Normally, computation of road surface roughness includes three steps: (1) data acquisition; (2) pre-processing; and (3) calculation. This paper chose the IRI as the evaluating indicator of road surface roughness. IRI is calculated from a measured longitudinal road profile by accumulating the output from a quarter-car model and dividing by the profile length to yield a summary roughness index with units of slope [12].

**Figure 2:**
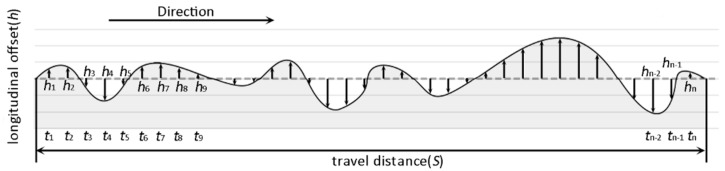
Example of a road longitudinal profile.

For example, for a vehicle driving on a road like that shown in Figure 2, $t_{i}\;\left(i\in \left[1,n\right]\right)$ is the sampling time, and *h_i_* is the longitudinal offset of the road surface at $t_{i}$. Through calculation, the vertical displacement ($Vh_{i}$) of each sampling interval can be obtained:

(1) $$Vh_{i}=\left|h_{i}-h_{i-1}\right|$$

Based on the IRI definition, we can sum up the vertical displacement of all sampling intervals, and divide it with the travel distance(*S*) to compute IRI:

(2) $$\mathrm{IRI}=\frac{\sum_{i=2}^{n}Vh_{i}}{S}=\frac{\sum_{i=2}^{n}\left|h_{i}-h_{i-1}\right|}{S}$$

Therefore, when computing IRS, we must know the total travel distance and the vertical displacement value of each sampling time. Travel distance can be computed via the GPS location. However, vertical displacement is not a value that can be obtained directly and can be derived from the output of the accelerometer sensors. In physics, we know that

(3) $$\begin{array}{c} \nu_{v}=\frac{dVh}{dt}\\ \alpha_{v}=\frac{d\nu_{v}}{dt}=\frac{d^{2}Vh}{dt^{2}} \end{array}$$

where *t* is time, $\nu_{v}$ is vertical speed, $\alpha_{v}$ is vertical acceleration, and Vh is the vertical displacement. Therefore

(4) $$\sum Vh=\iint_{t_{start}}^{t_{stop}}\left|\alpha_{v}\right|{\left(dt\right)}^{2}$$

Now, we can deduce that

(5) $$\mathrm{IRI}=\frac{\sum_{i=2}^{n}Vh_{i}}{S}=\frac{\iint_{t_{start}}^{t_{stop}}\left|\alpha_{v}\right|{\left(dt\right)}^{2}}{S}$$

**Figure 3:**
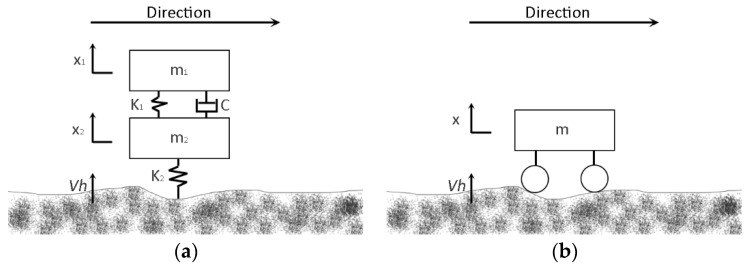
The original quarter-car model (**a**) and our bicycle model (**b**) used in the computation of the International Roughness Index (IRI).

Figure 3a shows the quarter-car model commonly used in computing IRI, in which the car can be divided into three parts: body, suspension and wheel. Several parameters are included when computing the road vertical displacement (*Vh*): the mass of the vehicle body (m_1_), the mass of wheel and suspension (m_2_), the elastic coefficient of the spring (K_1_), the elastic coefficient of the tire (K_2_), the damping coefficient of the suspension (C), and two body vertical displacements (x_1_ and x_2_). However, in the common bicycle structure, the frame’s and the wheel’s elastic capacities are very limited. Therefore, in the bicycle model (Figure 3b), the bicycle can be regarded as a rigid structure, so K_1_, C and K_2_ can be ignored; the model parameters are reduced to m and x. We assumed that the rider keeps the bicycle posture as stable as possible. In other words, he/she tries to avoid making the bicycle swing. To further reduce the impact of potential small swings, we used high-pass filter to clean the sensor data when we computed IRI (see Section 3.2.2 for more details). In the rigid structure, there is no spring shock effect and the bicycle body’s vertical displacement only makes contact with the tire–road surface, and therefore, x is equal to *Vh*.

**Figure 4:**
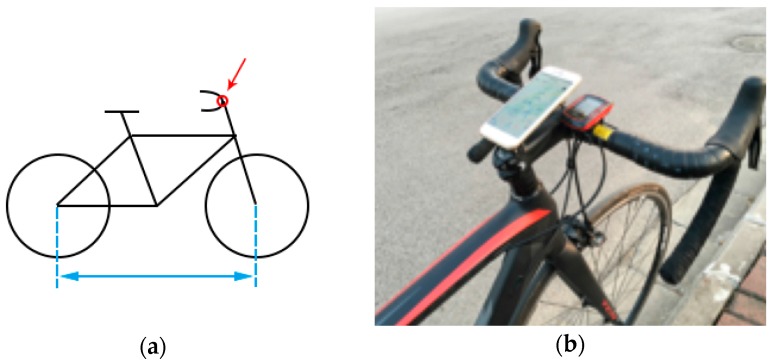
The installation position of smartphones on bicycles: (**a**) a sketch; (**b**) an example.

Another important thing that needs to be considered is the installation position of smartphones on bicycles. When the smartphone is fixed to different parts of a bicycle, different acceleration data might be recorded by the smartphone. Among all other places, the center of the handlebar seems to be a typical place for fixing smartphones on bicycles, according to cycling web platforms, like Cycling Weekly (http://www.cyclingweekly.com/) and ChinaBike (http://www.chinabike.net/). Therefore, in the current study, we fixed the smartphones to the center of the handlebar, as can be seen in Figure 4. We further discuss this issue in Section 5.

After these considerations, we are now ready to calculate IRI. As can be seen from Equation (5), we need to have *S*, $\alpha_{v}$, and *t* to compute IRI. In the next section, we explore how GPS sensors and accelerometer sensors on bicycle-mounted smartphones can be used to derive these parameters, and thus compute IRI.

### 3.2. Deriving IRI from GPS and Accelerometer Sensors on Bicycle-Mounted Smartphone

According to the analysis in the previous section, the basic elements of the IRI computing model are the accumulative vertical displacement value from the longitudinal profile (i.e., the numerator in Equation (5)) and the overall distance of the measurement (i.e., the denominator). Their values can be derived from sensors on bicycle-mounted smartphones. For example, the travel distance (*S*) can be computed using data recorded by GPS sensors on smartphones, and $\alpha_{v}$ can be obtained from accelerometer sensors. These two kinds of sensors are available in most of the current smartphones. GPS sensors on smartphones can return the current location (longitude/latitude) as well as information like travel speed. Accelerometers on current smartphones are often tri-axial, and provide acceleration information on three axes (*x*, *y*, and *z*) in the unit of g. In the following, we show how these data can be used to compute IRI, as specified in Equation (5).

#### 3.2.1. Calculating the Travel Distance (*S*)

There are two ways to calculate the travel distance (*S*). Firstly, with GPS, we can get the longitude and latitude coordinates of each sampling point. The travel distance can be computed by summarizing the distances between every two adjacent points. The distance between two sampling points can be calculated approximately using the Haversine formula, specifically,

(6) $$d=2*R*\arcsin\;\left(\sqrt{\sin^{2}\;\left(\frac{\varphi_{2}-\varphi_{1}}{2}\right)+\cos\left(\varphi_{1}\right)*\cos\left(\varphi_{2}\right)*\sin\left(\frac{\lambda_{2}-\lambda_{1}}{2}\right)}\right)$$

where $\varphi_{1}$ and $\lambda_{1}$ are the latitude and longitude of point 1, $\varphi_{2}$ and $\lambda_{2}$ are the latitude and longitude of point 2, and R is the Earth’s radius (mean radius = 6371 km).

Alternatively, the travel distance can be computed using the travel speed measured at each sampling point. Specifically,

(7) $$S=\int_{0}^{t}V_{t}dt$$

where $V_{t}$ is the travel speed measured at time t. It can be obtained directly from GPS sensors.

For smartphones, the latitude and longitude provided by GPS often lead to an accuracy of 10 m, while for instant travel speeds provided by GPS, the accuracies reported by manufacturers range from 0.1 m/s to 0.2 m/s [34]. Therefore, using travel speeds might lead to more accurate estimation of travel distance than using longitude/latitude coordinates of each GPS point [35]. Considering this, the second method was adopted in this paper to compute the travel distance.

#### 3.2.2. Obtaining Vertical Displacement

In theory, we can use altitude information returned by GPS for computing vertical displacement. However, the altitude accuracy of GPS sensors is often very poor, and their sampling frequency is often rather low (typically 1 Hz). Therefore, using altitude data provided by GPS for calculating vertical displacement is problematic. In this paper, we used vertical acceleration provided by accelerometers on smartphones, which allows a much higher sampling rate. As the installation of the smartphone on the bicycle as well as the orientation of the smartphone are unknown and variable, the vertical acceleration ($\alpha_{v}$) may appear in either dimension of the tri-axial acceleration data returned by the accelerometer. In other words, the *z*-axis acceleration data from the accelerometer cannot be taken directly as the vertical acceleration ($\alpha_{v}$). We need a method to derive $\alpha_{v}$ from the tri-axial acceleration values.

In the acquisition process, we set a requirement in advance: when the bicycle rider started the recording, the bicycle had to be in the normal riding posture, and kept stationary for more than 5 s. As the smartphone, which is mounted on the bicycle, is stationary, the only force it receives is the gravitational one, and the direction is vertical and downward, with a value of 1 g. Therefore,

(8) $$\bar{A_{x}}*\bar{A_{x}}+\bar{A_{y}}*\bar{A_{y}}+\bar{A_{z}}*\bar{A_{z}}=1$$

where $\bar{A_{x}}$, $\bar{A_{y}}$, and $\bar{A_{z}}$ are the average acceleration values of the *x*, *y*, and *z* axes in these 5 s, obtained from the accelerometer sensor on the smartphone. 

Deriving the vertical acceleration ($\alpha_{v}$) from any tri-axial acceleration output $A=\left(A_{x},A_{y},A_{z}\right)$ can be considered as projecting the vector A onto the reference vector $\bar{A}=\left(\bar{A_{x}},\bar{A_{y}},\bar{A_{z}}\right)$, measured at the beginning of the acquisition process. In other words, $\alpha_{v}$ is the scalar projection of vector A and $\bar{A}$. Therefore,

(9) $$\alpha_{v}=\frac{A\cdot \bar{A}}{\left|\bar{A}\right|}=\frac{A_{x}*\bar{A_{x}}+A_{y}*\bar{A_{y}}+A_{z}*\bar{A_{z}}}{\sqrt{\bar{A_{x}}*\bar{A_{x}}+\bar{A_{y}}*\bar{A_{y}}+\bar{A_{z}}*\bar{A_{z}}}}=A_{x}*\bar{A_{x}}+A_{y}*\bar{A_{y}}+A_{z}*\bar{A_{z}}$$

With these, the parameters required in Formula (5) have been fully prepared. We can then use it to compute IRI values from the data obtained from GPS and accelerometer sensors on smartphones.

During the acquisition process, we asked bicycle riders to keep the speed and the bicycle posture as stable as possible, and not to deliberately pass through or avoid bump areas on the road. To further reduce the influence of the potential speed changes and small swings, we used a high-pass filter (setting the value cutoff frequency as 0.5 Hz) to clean the sensor data when calculating vertical acceleration. We will further discuss these issues in Section 5.

#### 3.2.3. Sampling Frequency Selection

As showed in the above two sections, a GPS sensor and an accelerometer sensor are needed to estimate travel distance and vertical displacement. In order to provide accurate estimations, the sampling frequencies of both sensors should be carefully selected. For GPS sensors, we set the sampling frequency as 1 Hz. This is because the maximum update rate of GPS sensors on existing smartphones is 1 Hz (while professional GPS devices can offer higher frequencies). Setting the sampling frequency as 1 Hz allowed our method to be feasible for existing smartphones.

Regarding accelerometer sensors on smartphones, the sampling rate can go up to 2000 Hz. As mentioned in [12], the sample interval of IRI should be no larger than 300 mm for accurate calculation. The typical speed of bicycles is about 15.5 km/h [36]. Therefore, to allow accurate calculation of IRI, the sampling rate of accelerometers should not be less than 15.5 km/h ÷ 300 mm≈15 Hz. In order balance the sampling accuracy and the computational power of mobile phones, we chose 100 Hz.

### 3.3. Recognition of Potholes and Humps on Roads

In addition to measuring the road surface roughness (i.e., using IRI as in the above sections), we needed an algorithm to recognize potholes and humps on roads, and identify their locations. This kind of information is very useful for many applications, such as navigation and routing as well as road maintenance.

The underlying considerations of the algorithm are

(1) when a bicycle drives through a pothole or hump of the road, the accelerometer sensor on the bicycle-mounted smartphone will record some rather high instantaneous acceleration values. In other words, some spikes can be observed from the accelerometer output.
(2) The bicycle will touch the pothole or hump twice, once with the front wheel and the other time with the rear wheel. Therefore, at least two spikes will be observed. Furthermore, the duration between the first spike and the last one is longer than L/ν, where L is the distance between the centers of the front and rear wheels, and ν is the travel speed of the bicycle.
(3) If two adjacent potholes or humps are longer than L, we would consider that these two adjacent potholes or humps are far away from each other and should be considered as two different potholes or humps.

**Figure 5:**
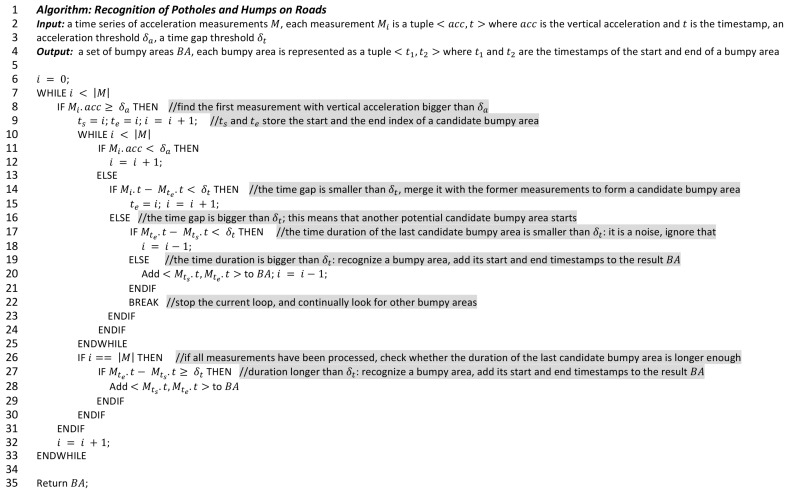
Algorithm for the recognition of potholes and humps on roads.

Figure 5 shows the algorithm in pseudo code. It takes a series of vertical acceleration measurements, an acceleration threshold ($\delta_{a}$), and a time gap threshold ($\delta_{t}$) as inputs. The output of the algorithm is a set of bumpy areas, containing the timestamps of the start and the end of each of the bumpy areas. These timestamps can be synchronized with GPS measurements to identify the locations of these bumpy areas. The acceleration threshold ($\delta_{a}$) is used to recognize spikes from the acceleration measurements, while the time gap threshold ($\delta_{t}$) is used to check the time distance between spikes (as mentioned before). The algorithm tries to find the first measurement with a vertical acceleration bigger than $\delta_{a}$ (Line 8), i.e., the first spike. When the first spike is found, it continually searches for the next one. If the time difference of a spike and its previous one is bigger than $\delta_{t}$, a candidate pothole or hump area with all the previous spikes is formed (Lines 10–15). If the duration of the candidate area is bigger than $\delta_{t}$, this candidate area is recognized as a pothole/hump area (Lines 17–21). Otherwise, it is a noise. The algorithm continues this process until the last measurement. At the end, it returns a set of bumpy areas.

The algorithm has two parameters to calibrate: the acceleration threshold ($\delta_{a}$), and the time gap threshold ($\delta_{t}$). In our initial test, we found that when riding at normal speed, even on rough roads, the instantaneous vertical acceleration value did not exceed 2 g. Therefore, we set the value of $\delta_{a}$ to 2 g. The time gap threshold ($\delta_{t}$) can be set as L/ν, where L is the distance between the centers of the front and rear wheels, and ν is the travel speed of the bicycle. Therefore, it can be computed automatically based on the travel speed of the bicycle, e.g., as derived from GPS sensors. In our evaluation experiments, we set $\delta_{t}$ as 1 m÷15.5 km/h≈0.23 s, as the distance between the centers of the front and rea wheels of adult bicycles is normally 1 m, and the typical speed of bicycles is about 15.5 km/h [36].

## 4. Implementation and Evaluation

As a proof of concept, this section describes the implementation of a mobile application for mapping road surface roughness and identifying potholes and humps on roads, using the method and algorithm introduced above. We further evaluate the proposed methodology with three experiments, aiming to answer the following questions: (1) Are the IRI values measured comparable to those reported with professional instruments? (2) How do the IRI values measured differ from different bicycles, different people and the fixing methods of smartphones? (3) How well does the algorithm perform in recognizing potholes and humps on roads?

### 4.1. Implementation

We developed a mobile phone application called “RoadSR” based on an iOS system. The application uses GPS and accelerometer sensors on smartphones to record location and acceleration information. The sensor outputs are then further processed to calculate IRI values as well as to identify potholes and humps on roads. The application finally visualizes the IRI values computed and the potholes and humps are identified on a map, using AutoNavi map SDK.

**Figure 6:**
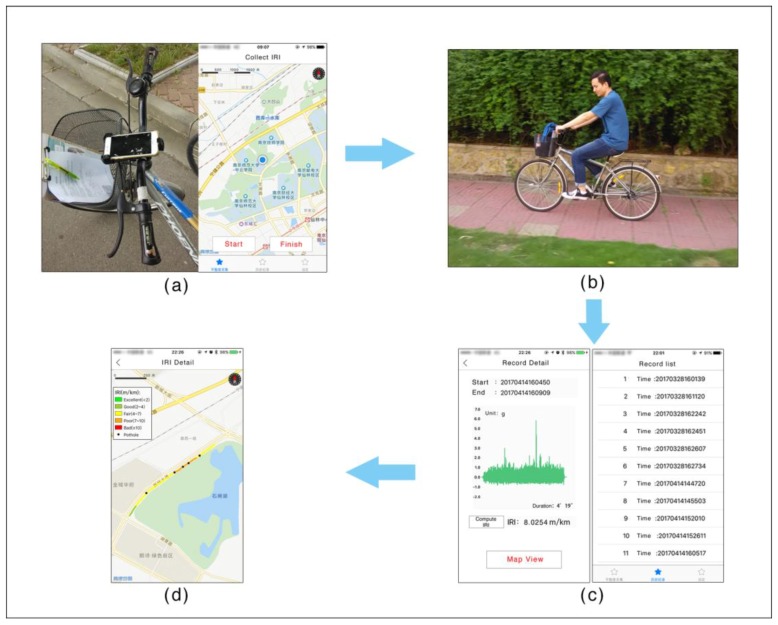
Measuring process based on the “RoadSR” app. (**a**) The rider fixes the smartphone onto the bicycle and clicks “Start” to start data recording. (**b**) The rider rides the bicycle. (**c**) When the recording is stopped, the app automatically calculates the IRI values of the road just traveled and identifies potholes/humps. (**d**) The computed IRI values and the potholes/humps recognized are visualized on a map view.

To use “RoadSR”, the rider only needs to fix the smartphone onto the bicycle, click the “Start” button on the bottom left of the application interface, and data about the road surface roughness will be automatically collected using GPS and accelerometer sensors (Figure 6a). The rider can click the “Finish” button on the bottom right to stop recording. When the recording is stopped, the application automatically calculates the IRI values of the road just traveled and identifies potholes/humps (Figure 6c). The rider can then view the results on a map view. Figure 5d shows a map view, visualizing the bicycle trajectory, the surface roughness (IRI) of the road in different colors, and potholes/humps along the route as black dots.

### 4.2. Experiment 1: Comparing the Method with the Professional System

**Figure 7:**
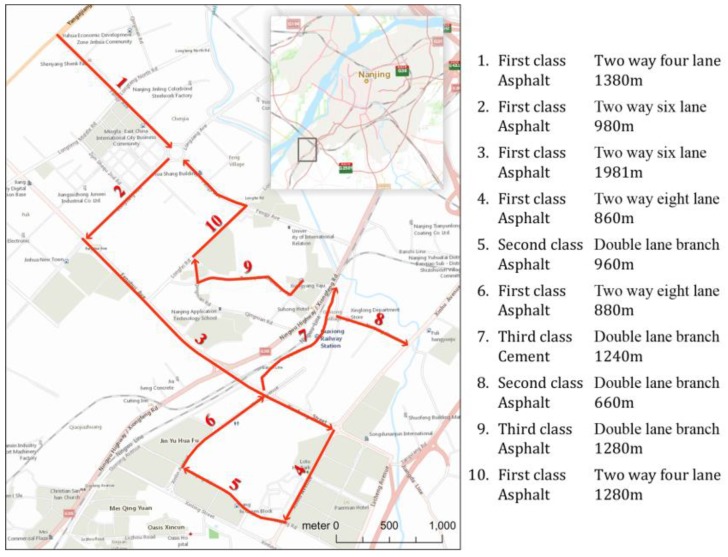
The 10 test roads of Experiment 1 and their details, located in Banqiao Town, southwest of Nanjing, China.

This experiment aimed to study how well the IRI values measured by the proposed method correlate with those measured by professional instruments. We selected 10 road sections in Banqiao Town, southwest of Nanjing, China (Figure 7). These 10 road sections differed in pavement materials and conditions, road widths and lengths. We used the car mounted laser pavement scanner (see Figure 1) from the Nanjing Highway Scientific Research Institute, which is a professional instrument and system for measuring IRI values on motorable roads. For each road section, we first drove the car, followed by the bicycle. When riding the bicycle, we tried to keep the speed and the bicycle posture as stable as possible. At the end of the collection, we computed the IRI values of each road section, taking 20 m as the computing unit.

A Pearson correlation coefficient was computed to assess the relationship between the IRI values measured by the car with the laser scanner to those measured by our proposed method. The results show that there was a very positive and significant correlation between the IRI values measured by the proposed method and those measured by professional instruments (*r* = 0.893, *n* = 9, *p* = 0.001).

In summary, the experiment shows that the IRI values measured by the proposed method strongly and positively correlate with those measured by professional instruments. In other words, the proposed bicycle-mounted smartphone based IRI collection method can well reflect the road roughness level. 

### 4.3. Experiment 2: Method Stability

**Figure 8:**
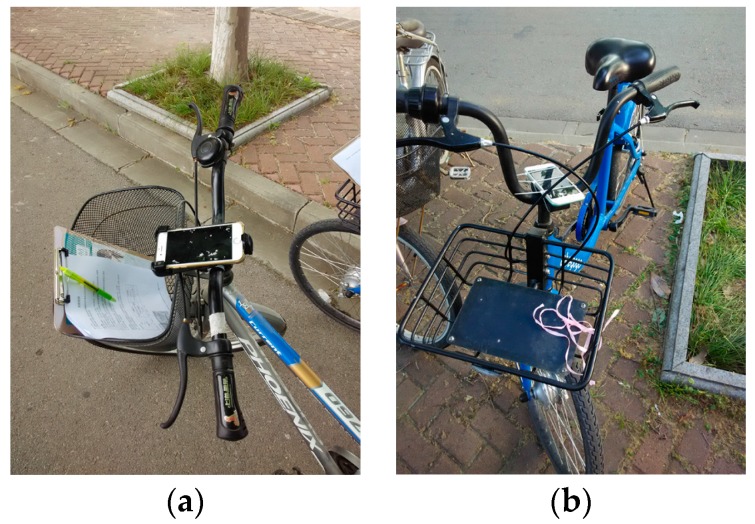
Photos of the two sets of equipment and smartphone fixing modes: (**a**) Group A: mountain bicycle with iPhone 6S, with fixing holder; (**b**) Group B: “Bluegogo” sharing city bicycle with iPhone 6, directly fixed.

This experiment aimed to investigate how the IRI values measured by the proposed method differ for various people, bicycles and smartphone fixing methods. Particularly, we used two different settings (Figure 8): Group A (mountain bicycle + fixing holder), and Group B (city bicycle + directly fixed). We assigned three male graduate students to each group. We told them to ride on the right side of the road and to not deliberately pass through or avoid bumpy areas on the roads.

**Figure 9:**
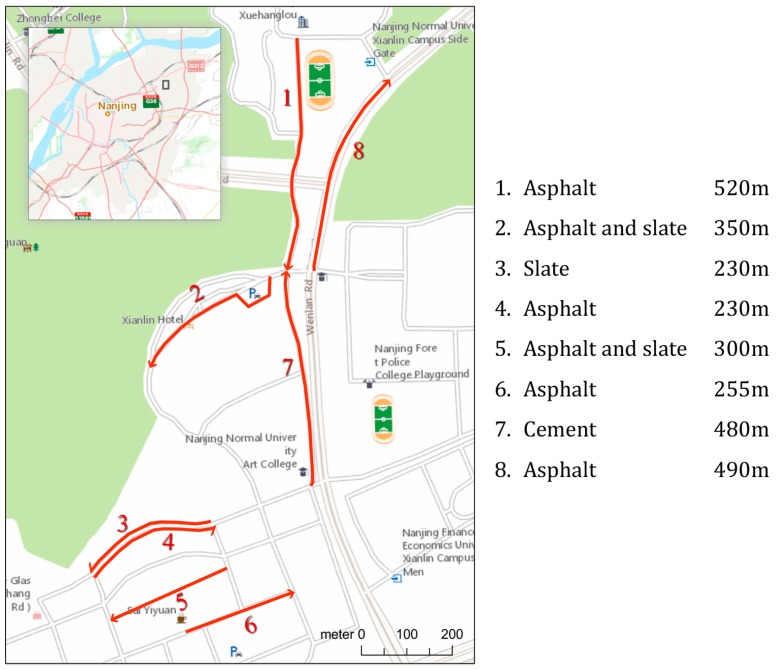
The eight test roads of Experiment 2 and their details, located in the Xianlin campus of Nanjing Normal University, Nanjing, China.

The experiment was carried out on eight road sections in the Xianlin campus of Nanjing Normal University (Figure 9). Each road section was measured by two sets of equipment (Group A, Group B), each with three users. In total, there were six data collections for each section. We also ensured that each user only rode one time in each section.

**Figure 10:**
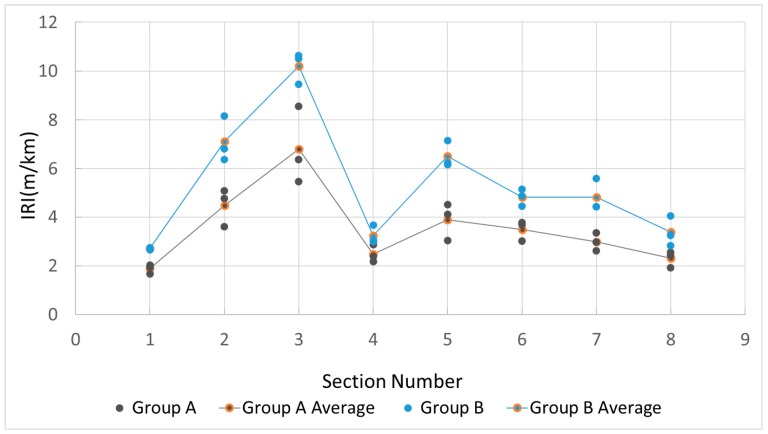
Comparison of IRI values measured by different groups and users.

Figure 10 shows the results of the experiment. Several interesting results can be seen from Figure 10. Overall, the IRI values measured by both groups fell into the same range, with an average difference of 1.80 m/km; Meanwhile, the IRI values of group A were always lower than those of group B. (2) Within the same group, IRI values measured by different users seem rather consistent. The average difference in Group A was 0.76 m/km, while in Group B it was 0.66 m/km. In other words, the deviation over different users seems much smaller than the deviation over different groups (A and B). (3) The rougher the road, the greater the IRI deviation between users and groups.

In summary, this experiment shows that overall the proposed bicycle-mounted smartphone based IRI values measured by different sets of equipment and different users, to some extent, are very consistent, with a rather small average difference of 1.80 m/km. Meanwhile, the proposed method achieves a very good consistency over different users, better than that with different equipment. More research should be done to investigate these results further.

### 4.4. Experiment 3: Recognition of Potholes and Humps on Roads

**Figure 11:**
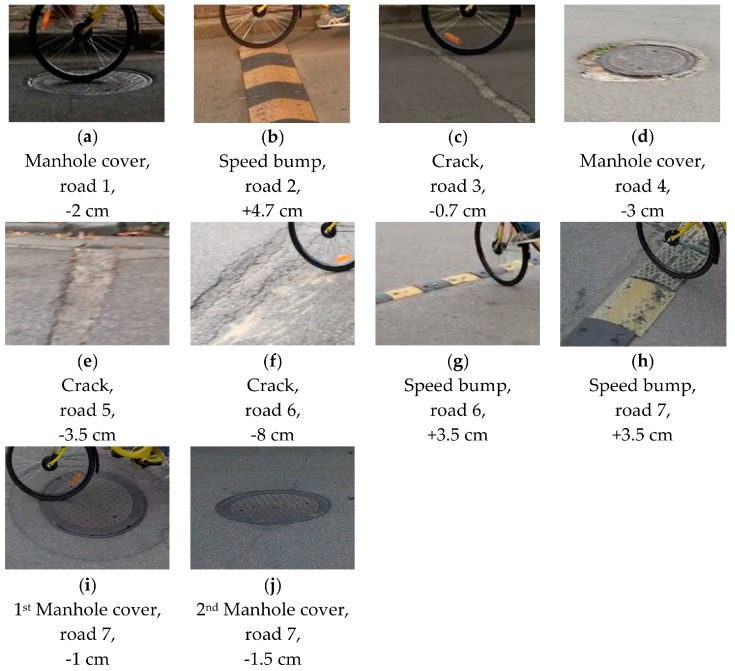
Potholes and humps on the seven road sections, as well as their types and longitudinal offsets.

This experiment aimed to evaluate the performance of the potholes/humps recognition algorithm proposed in Section 3.3. We chose seven road sections with different types of potholes and humps (Figure 11). The potholes and humps differed in type and longitudinal offset. We started the application and rode on these road sections. In order to record the actual riding tracking and the potholes/humps that the bicycle passed through, we used a tracking camera mounted on an unmanned aerial vehicle (UAV) to capture the image data at the moment that the bicycle passed a pothole/hump.

**Figure 12:**
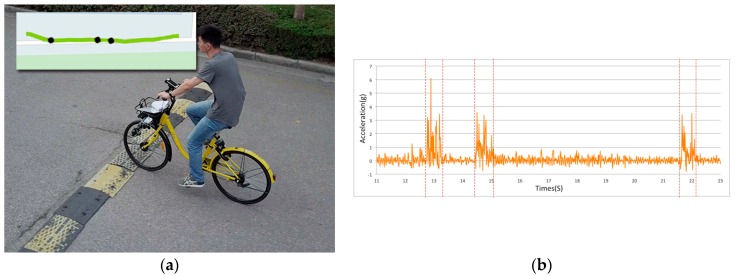
Potholes and humps at Road Section 7 and the accelerometer data. (**a**) The test bicycle was ridden over the speed bump and the other two humps (i.e., manhole covers), all of which were correctly recognized by the proposed algorithm; (**b**) the accelerometer data recorded when the bicycle was ridden over these areas.

**Figure 13:**
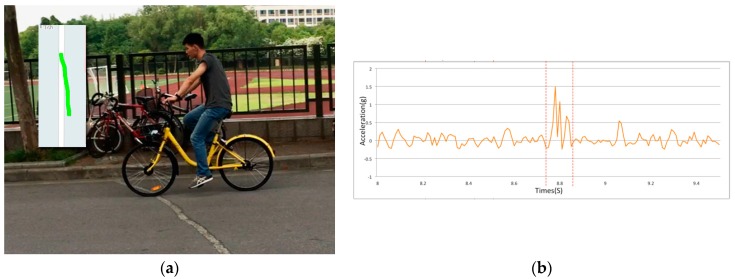
Crack at Road Section 3 and the accelerometer data. (**a**) The test bicycle was ridden over the crack which the proposed algorithm failed to recognize; this is due to the fact it is rather small and its longitudinal offset is only about −0.7 cm; (**b**) the accelerometer data recorded when the bicycle was ridden over this area.

We compared the potholes/humps recognized by the proposed algorithm with the ground truth obtained from the UAV image data. The results of the experiment show that the proposed algorithm was able to correctly recognize all of the potholes/humps (and their locations) that the bicycle passed (see Figure 12 for an example), except for the crack in the third road section (Figure 13). Having a closer look at the crack, we found that it is rather small, and its longitudinal offer is about −0.7 cm, which is the main reason why the proposed algorithm failed to detect this crack. This raises an interesting question regarding what can be considered as a pothole or hump, or more precisely, a pothole or hump that influences peoples’ travel experiences. More research should be done on this aspect.

## 5. Discussion

As shown in the above section, “Implementation and Evaluation”, the proposed method is feasible for measuring road surface roughness and identifying potholes/humps on roads, using data recorded by GPS and accelerometer sensors on bicycle-mounted smartphones. The three experiments illustrated that the IRI values measured by the proposed method strongly and positively correlate with those measured by professional instruments. Meanwhile, the proposed algorithm was able to recognize the potholes/humps (and their locations) that a bicycle passed. The proposed method is very useful for measuring surface roughness of roads that are not accessible for professional instruments (which are often installed on motor vehicles), such as pedestrian lanes and cycle lanes.

When measuring the road surface roughness using the proposed method, we asked bicycle riders to keep the speed and the bicycle posture as stable as possible, and to not deliberately pass through or avoid bump areas on the roads. This might not be very realistic for real-world settings, as users might change their posture during riding. Changing the bicycle posture will influence the acceleration signals of the accelerometer sensor, and thus have a negative impact on the measurement results. Methods regarding signal processing and noise removal might help to address this issue. We will address this in our follow-up studies.

In this study, we fixed smartphones on the center of the bicycle handlebar. While this is a typical place where cyclists fix their smartphones, it might be interesting to investigate whether different installation positions on the bicycle lead to different measurement results. To comprehensively address this issue, the installation position of the smartphone should be considered together with the length of the wheelbase of the bicycle. In some extreme cases, the accelerometer sensor on the smartphone might be only subjected to angular acceleration. Therefore, it makes sense to investigate this issue in detail, and identify an optional installation position based on the length of a wheelbase.

In Experiment 1, we compared the IRI values measured by a professional vehicle and those by our proposed bicycle-mounted smartphone-based method. It is important to note that the vehicle travelled in the center of the roads, while for security reasons, the bicycles only used the side of the road. Therefore, the travel trajectories do not completely match. This obviously led to some differences in the IRI values they computed. To further improve this experiment, one could use the bicycle on the other side of the roads that the vehicle traveled, and check whether the average IRI values match those from the professional vehicle.

Experiment 2 showed that different bicycles (i.e., mountain bicycle and city bicycle) lead to slight differences in the IRI values measured. This might be due to the different elasticity coefficients of the bicycles and their wheels/tires. Further studies should be done to investigate this aspect further. Meanwhile, it would also be interesting to see whether different riding styles lead to different measurement results.

The proposed algorithm for identifying potholes/humps currently uses a fixed acceleration threshold ($\delta_{a}$) (we set it as 2 g for the experiments) to identify sparks in the signal data. While the algorithm and the threshold value worked very well, as shown in the experiment results, the algorithm could be further improved to make it a self-adaptive algorithm.

An important implication of this work should be also mentioned. As shown in the experiments, the proposed bicycle-mounted smartphone-based method can be used to map the road surface roughness and identify potholes/humps on roads. Currently, cycling in cities is becoming more and more popular. Therefore, potentially a huge number of contributors are available to map the road surface roughness around the areas they travel, using their own smartphones. This would be a nice add-on to the VGI project, OpenStreetMap. Having this kind of information available would allow many interesting and innovative applications, such as navigation and routing for pedestrian, bicycles and wheelchairs.

## 6. Conclusions and Future Work

Recent years have seen increasing interest in mapping the surface roughness of roads, as it is closely related to driving safety, passenger comfort, road maintenance and so on. While several professional systems, such as vehicle-mounted laser scanning, exist, they can only be used for motorable roads. Until now, there has been no effective method for measuring the road surface roughness of pedestrian and bicycle lanes. This article proposed a method for measuring the road surface roughness of pedestrian and bicycle lanes based on GPS and accelerometer sensors on bicycle-mounted smartphones. We mainly focused on the IRI metric.

A proof-of-concept and three experiments were designed to evaluate the proposed method. Results of the implementation and the experiments showed that the IRI values measured by the proposed method strongly and positively correlate with those measured by professional instruments, and the proposed method was able to recognize potholes and humps (and their locations) passed by the bicycles.

In conclusion, bicycle-mounted smartphones can be used to measure the surface roughness of roads as well as to identify potholes and humps along the roads. This approach is especially useful for those roads that are not accessible by professional instruments (which are often installed on motor vehicles like cars), such as pedestrian lanes and cycle lanes. This work enables us to further study the feasibility of large-scale crowdsourcing of road surface roughness (like OpenStreetMap) with bicycle-mounted smartphones, as cycling has become more and more popular in recent years.

As a next step, we will further improve the proposed method by considering different bike-riding styles, travel speeds, bicycle models, smartphone installation positions, and road conditions (e.g., uphill and downhill). In the meantime, we would like to develop a self-adaptive algorithm to identify potholes and humps on the roads. Furthermore, as mentioned previously, we would like to expand this work towards large-scale crowdsourcing of road surface roughness. To achieve this aim, we plan to implement a web platform allowing cyclists to upload their recorded GPS and accelerometer data, as well as provide some other information, like bike type and relevant parameters (e.g., length of wheelbase), and installation position of smartphones. The IRI values will then be derived, stored, and visualized in a map view. Algorithms will then be developed to combine results from different cyclists for the same road segment. These IRI values can be also integrated to enrich the VGI project, OpenStreetMap, with surface roughness information on pedestrian and cycle lanes. This will allow many interesting applications, such as navigation and routing for bicycles, pedestrian, and wheelchairs, and road maintenance.
